# Earthquakes in 1881

**Published:** 1882-07

**Authors:** 


### EARTHQUAKES IN 1881.

Two hundred and forty-four earthquakes, it is stated, are known to have occurred during 1881, of which 86 were in winter, 61 in autumn, 56 in spring, and 41 in summer. The earthquake period at Agram, beginning in November, 1880, extended into 1881, in which earth vibrations were observed on twenty-four days, many of the shocks being very violent. Among the great earthquakes of 1881 that of Chios takes the first place. The tremendous first shock in the afternoon of April 3d laid most of the town of Castro in ruins. This earthquake, lasting six days in full strength (with thirty or forty very violent shocks), was felt most in the southern part of the island. On the mainland, the port of Tschesme was half destroyed. In Chios, 4181 persons were killed and about 1000 injured. From April 10th the phenomena gradually abated; but strong shocks occurred on May 20th, June 10th, August 27th and even in the end of November. The violent earthquake of Ischia (March 4) caused the death of about 150 persons; it was quite local—confined to the district of Casamicciola and Lacco. A loud noise about 1:05 P.M. was followed by the shock which, lasting seven seconds, wrought most of the ruin; the movement seemed undulatory and jerky, and threw down whole streets in the upper part of Casamicciola; in the lower part, and in Lacco, over the hill, only a few houses suffered. A second weaker shock occurred at 4 P.M. The fine seismographs at Vesuvius Observatory and at Naples gave no sign.

Other notable earthquakes occurred at Osogna in Abruzzo on August 10th, ruining about 1000 houses; between Tabreez and Khoi, from

August 28th to September 11th ; and at the Azores, from the end of February extending into March ; this last was connected with a submarine eruption, and in San Miguel destroyed 200 houses. Some interesting seismic phenomena occurred in Switzerland ; the basin of the Lake of Geneva is indicated as a chief centre of vibration, whence principally Western Switzerland is affected. The more violent shocks extended into France or the Black Forest ; neither Alps nor Jura proved an obstacle. Another centre appeared on November 18th in East Switzerland, between Santis and Glarnisch, and the effects reached to the Tyrol, the Southern Black Forest, the Jura and the Ticino. Vorarlberg seems to have been the seat of independent earthquakes, with the Arlberg as centre. On January 10th there was an earthquake on the eastern side, and on December 2d one on the western ; and on November 5th a movement extended from Arlberg over the Bergenzerwald, most of East Switzerland, and as far as Zurich. In the flat regions of the Danube three earthquakes were observed (February 5th and 11th and April 3d). In Belgium there was a small earthquake on February 28th, in Beckarth and Wickrath ; and a stronger one occurred on November 18th, affecting Belgium, the Rhine provinces and Westphalia, and having its centre about Charleroi. Saxony had earthquake motions on May 22d and September 24th.

